# Feasibility of Tomotherapy-Based Image-Guided Radiotherapy for Small Cell Lung Cancer

**DOI:** 10.3389/fonc.2013.00289

**Published:** 2013-11-26

**Authors:** Nam P. Nguyen, Wei Shen, Sarah Kratz, Jacqueline Vock, Paul Vos, Vinh-Hung Vincent, Gabor Altdorfer, Lars Ewell, Siyoung Jang, Ulf Karlsson, Juan Godinez, Melissa Mills, Thomas Sroka, Suresh Dutta, Alexander Chi, The International Geriatric Radiotherapy Group

**Affiliations:** ^1^Department of Radiation Oncology, University of Arizona, Tucson, AZ, USA; ^2^Division of Pulmonary Medicine, University of Arizona, Tucson, AZ, USA; ^3^Division of Hematology Oncology, University of Arizona, Tucson, AZ, USA; ^4^Department of Radiation Oncology, Lindenhofspital, Bern, Switzerland; ^5^Department of Biostatistics, East Carolina University, Greenville, NC, USA; ^6^Department of Radiation Oncology, University of Geneva, Geneva, Switzerland; ^7^Department of Radiation Oncology, Camden Clark Medical Center, Parkersburg, WV, USA; ^8^Department of Radiation Oncology, Marshfield Clinic, Marshfield, WI, USA; ^9^Department of Radiation Oncology, Florida Radiation Oncology, Jacksonville, FL, USA; ^10^Department of Radiation Oncology, Darmouth College, New Lebanon, NH, USA; ^11^Medicine and Radiation Oncology PA, San Antonio, TX, USA; ^12^Department of Radiation Oncology, West Virginia University, Morgantown, WV, USA

**Keywords:** small cell lung cancer, limited stage, chemoradiation, IGRT

## Abstract

**Background**: To assess the tolerance of patients with small cell lung cancer undergoing chemoradiation with tomotherapy-based image-guided radiotherapy (IGRT).

**Materials and Methods**: A retrospective review of the toxicity profile for nine patients with small cell lung cancer of the limited stage who underwent chemoradiation delivered with helical tomotherapy (HT) has been conducted.

**Results**: Acute grade 3–4 hematologic and esophagitis toxicities developed in two and three patients respectively. One patient developed a pulmonary embolism during radiotherapy. Seven patients had weight loss ranging from 0 to 30 pounds (median: 4 pounds). Three patients had treatment breaks ranging from 2 to 12 days. At a median follow-up of 11 months (range: 2–24 months), no patients developed any radiation related toxicities such as grade 3–4 pneumonitis or other long-term complications. The median survival was estimated to be 15 months. There were two local recurrences, three mediastinal recurrences, and six distant metastases.

**Conclusion**: Grade 3–4 toxicities remained significant during chemoradiation when radiation was delivered with tomotherapy-based IGRT. However, the absence of grade 3–4 pneumonitis is promising and the use of HT needs to be investigated in future prospective studies.

## Introduction

Standard of care for small cell lung cancer of limited stage is concurrent chemotherapy and radiotherapy (1–7). Radiotherapy usually starts within 30 days of the initiation of chemotherapy to ensure an optimal outcome (5). Treatment toxicity of the combined modality is significant because of the volume of the normal organs irradiated (esophagus and lungs) and the bone marrow suppression from chemotherapy. Grade 3–4 esophagitis, pneumonitis, and hematologic toxicity occurred frequently (1–7). Although there are still controversies about the technique of radiation delivery and the most optimal dose of radiotherapy, most institutions treated the gross tumor and mediastinum, which is followed by a cone down to the gross tumor with the three-dimensional conformal radiotherapy technique (3-D CRT). Some institutions choose to treat the gross tumor and involved lymph nodes only to decrease normal tissue toxicity with the risk of recurrence in the non-irradiated mediastinal lymph nodes (4). Recently, new techniques of radiotherapy such as image-guided radiotherapy (IGRT) has been introduced to help reduce the treatment margins used to create the planning target volume through more precise image guidance. This potentially decreases normal tissue toxicity through reducing the volume of normal tissue included in the high dose volume. It also allows improved tumor coverage and the delivery of higher radiation to the target volume which potentially improves loco-regional control without increasing the risk of normal tissue complications. Preliminary results of IGRT in head and neck and gastrointestinal cancers have been promising because of significant sparing of normal organs at risk for radiotherapy complications (8–12). This has prompted us to conduct this retrospective study on the feasibility of delivering IGRT with helical tomotherapy (HT) in the setting of concurrent chemoradiation for limited-stage small cell lung cancers.

## Materials and Methods

The medical records of nine patients with limited-stage small cell lung cancers who underwent concurrent chemoradiation delivered with HT between September 2009 and February 2012 were retrospectively reviewed. The University of Arizona Institutional Review Board (IRB) approved the study and waived the requirement for patient consent because of the retrospective nature of the study. All but one patient underwent platinum-based chemotherapy with cisplatinum and etoposide (7) or carboplatin and etoposide (1). One patient refused chemotherapy because of the fear of treatment-related toxicities. All patients had mediastinal nodal involvement (8 N2, 1 N3) at presentation. Six patients had stage IIIA and three patients had stage IIIB disease. Table 1 summarizes patient characteristics. All patients underwent positron emission tomography (PET) with computed tomography (CT) as part of the staging. The PET-CT was also incorporated into the planning CT to outline the target volume. All patients underwent simulation and treatment in the supine position with their arms raised above their heads and were immobilized using a custom-made Vac-Lok cradle (Medtec, Orange City, IA, USA). For the actual simulation, a CT scan of the chest with and without intravenous (IV) contrast was performed in the treatment position. The thorax area from the neck to mid-abdomen was scanned with a slice thickness of 3 mm. The CT scan without contrast was used for planning to avoid possible interference of the contrast density on radiotherapy isodose distribution. The gross tumor volume (GTV) was defined as the post-chemotherapy tumor volume after one cycle of chemotherapy. All patients underwent 4D simulation at the time of the CT simulation to outline the tumor internal target volume (ITV). Respiratory motion was accounted for by 4D CT which retrospectively sums of the CT images collected from evenly separated time points (10 phases) throughout one breathing cycle. The clinical target volume (CTV) was created by expansion of the ITV, PET positive mediastinal lymph nodes, ipsilateral hilum, and mediastinum with a 0.8 cm margin. For tumor located in the upper lobes (8), the ipsilateral supraclavicular lymph node area was also treated prophylactically and the lower mediastinum was excluded. For tumor located in the lower lobe, the lower mediastinum was treated but the supraclavicular lymph node area was excluded. A margin of approximately 0.2 cm was added to create the final PTV. The PTV and ITV was treated to a total dose of 4500 cGy in 180 cGy/fraction and 5000 cGy in 200 cGy/fraction respectively. All target volumes were checked daily before each treatment and the isodose lines verified to ensure that no excessive radiation was delivered to the OARs. Patients were simulated and re-planned at 4000 cGy to account for tumor shrinkage, and/or expansion of the lungs from resolution of the atelectasis. The residual gross tumor ITV was boosted for an additional dose of 1000 cGy in 200 cGy/fraction (6) or 1500 cGy in 125 cGy/fraction twice a day (bid) (3) to achieve a tumor dose of 6000–6500 cGy. The bid fractionation was chosen when the tumor encased the blood vessels to decrease the risk of hemorrhage as the tumor may already invade into the adventitia of the blood vessels. Dose constraints for normal organs at risk for complications were the following: lungs (V20 <30%, V5 <50%); spinal cord: *D*_max_ <40 Gy; cardiac ventricles: V40 <10%, V20 <50%). Minimal target coverage was 95% for all targets with at least 99% of the prescribed dose delivered to the gross tumor and mediastinal lymph nodes. Radiotherapy started on the second cycle of chemotherapy. Weekly complete blood count (CBC) and blood chemistry to assess renal function were performed during chemoradiation.

**Table 1:** **Patient characteristics**.

Patient number	9
Median age	60 (46–84)
Sex
Female	2
Male	7
Histology	Small cell
T stage
T1	4
T2	3
T4	2
N stage
N2	8
N3	1
Stage
IIIA	6
IIIB	3
Tumor location
RUL	4
LUL	3
RML	1
LLL	1
Follow-up	2–24 months (median: 11 months)

*RUL: right upper lobe; LUL: left upper lobe; RML: right middle lobe; LLL: left lower lobe*.

Treatment breaks and weight loss were recorded during chemoradiation. Acute and long-term toxicities were graded according to Radiotherapy Oncology Group (RTOG) group severity scale (http://ctep.cancer.gov). All patients had a follow-up visit 1 month and regularly 3 months following treatment. Clinical examination was performed at each follow-up to detect recurrent disease and possible complications resulting from the treatment. Patients were asked specifically about their exercise tolerance, dysphagia, weight loss, and difficulty to breathe compared to their pre-treatment baseline. The patients were also monitored during and following treatment with a team of dietitians because of the expected grade 3–4 esophagitis. A PET-CT scan was performed 4, 10 months, and yearly after treatment if there was no clinical evidence of disease. Patient with a complete response (CR) on PET-CT will undergo prophylactic cranial irradiation (PCI). Survival data was analyzed using Kaplan–Meier estimation.

## Results

PTV target coverage ranged from 96 to 98% (median: 97%). Maximum spinal cord dose ranged from 3300 to 4100 cGy (median: 3500 cGy). V20 and V5 for both lungs combined ranged from 6 to 25% (median: 22%) and 30 to 67% (median: 53%) respectively. Because of the tumor location in the upper lobes in eight patients, the cardiac ventricles doses were insignificant. Except for one patient, all patients had significant shrinkage of the GTV during treatment (30% or more of the pre-treatment tumor volume). For one patient, tumor size increased during treatment and he developed loco-regional failure later.

Two patients (22%) developed grade 3–4 hematologic toxicity. Three patients (33%) had grade 3–4 esophagitis and one patient (11%) had pulmonary embolism. Median weight loss was 4 pounds (range: 0–30 pounds). Three patients (33%) had treatment breaks ranging from 2 to 12 days (Table 2). Four patients (44%) had a CR on PET-CT post treatment. However, only one patient with a CR underwent PCI. The other three declined PCI because of the fear of late neurotoxicity. Two of these patients developed multiple brain metastases later and died. Two patients developed local recurrences (22%) and three patients had mediastinal recurrences (33%). Six patients (66%) developed distant metastasis (brain: 2, liver: 2, bones: 2, adrenals: 1). At a median follow-up of 11 months (range: 2–24 months), the median and 2-year survival was estimated to be 15 months and 28% respectively. No patients developed grade 3–4 acute or late pneumonitis. The acute esophagitis resolved by 6 weeks following treatment completion and all patients recovered from their weight loss. No patient noticed a change of their breathing pattern or physical activity compared the pre-treatment baseline.

**Table 2:** **Treatment toxicity**.

Weight loss	0–30 pounds (median: 4 pounds
Treatment break	Three patients had treatment breaks ranging from 2 to 12 days
**Grade 3–4 toxicities**
Hematologic	2/9 (22%)
Esophagitis	3/9 (30%)
Pulmonary embolism	1/9 (11%)

## Discussion

Randomized studies of small cell lung cancer with limited stages revealed poor survival with a high rate of loco-regional recurrences and distant metastases (2, 6, 13–17). Radiation therapy dose ranged from 4500 cGy in 150 cGy bid to 4500–5000 cGy in 25 daily fractions. Despite a low dose of radiation, patients still experienced grade 3–4 pneumonitis ranging from 3.2 to 6.2% (2, 13, 15, 16). In severe cases death may occur (2, 13, 15). Among long-term survivors, grade 3–4 pulmonary fibrosis has been reported in 37–39% of the patients (17). Radiation-induced lung injury was most likely due to irradiation of a large volume of the normal lungs with the conventional radiotherapy technique when the ipsilateral mediastinum was included in the treatment volume. Preliminary data indicated that by limiting the target volume to the GTV and adjacent lymph nodes, radiation dose escalation was feasible to improve local control. With this approach, Bogard et al. reported that the GTV dose may be increased to 7000 cGy with grade 3 pneumonitis observed in only 5% of the patients (18). Thus, a new radiation technique that allows radiation dose escalation without increasing lung toxicity may potentially improve local control and survival while preserving patient quality of life. The introduction of intensity-modulated radiotherapy (IMRT) may allow for decrease normal tissue toxicity because of the rapid dose fall-off away from the target volume (19). However, when IMRT was used for elective nodal irradiation in patients with limited-stage small cell lung cancer, 7% of the patients still experienced grade 3 pneumonitis despite the fact that two-third of the patients was treated to 4500 cGy in 30 bid fractions and the target was limited to the GTV and lymph nodes involved on PET scan (20). Thus, our study is the first to demonstrate the feasibility of adaptive IGRT delivered with HT-based image to spare the normal lungs from excessive irradiation as the GTV was treated to a higher dose of radiation (6000–6500 cGy) and the ipsilateral mediastinum was included in the treatment field. The absence of grade 3–4 pneumonitis and long-term lung injury in our study may be attributed to multiple factors. We outlined the GTV post-chemotherapy as a target volume as Hu et al. (3) observed no difference in local control if the post-chemotherapy GTV was treated instead of the pre-chemotherapy GTV. As the tumor shrinks during radiotherapy, re-planning and boosting to the residual tumor may allow for sparing of the normal lungs without compromise of target coverage (21). All but one of the patients had significant decreased in size of the tumor which is in agreement with other studies. Hugo et al (22) reported that the GTV volume decreased by 23% after 5 weeks of radiotherapy in patients with non-small cell lung cancer. In addition, compared to conventional IMRT, HT provides comparable target coverage with a significant reduction of the lungs V20 (23). We also set high priority to limit the V5 for normal lung as the patients received concurrent chemotherapy which may increase radiosensitization of the normal lungs and the risks of severe pneumonitis (23). The low V5 and V20 in our study indicated that the normal lungs may be preserved from excessive irradiation with HT-based IGRT in the treatment of patients with small cell lung cancer of limited stage. This is true even if elective nodal irradiation is administered. Thus, dose escalation to the gross disease with reduced margins may be feasible with HT-based IGRT. This hypothesis warrants further validation in future prospective trials with larger number of patients.

We do observe a high rate of grade 3–4 esophagitis which is unavoidable because the esophagus is included in the ipsilateral mediastinum. However, all patients recovered following treatment and resumed a normal physical activity. The addition of a radiation protector such as amifostine may be an option to decrease the severity of esophagitis and improve patient quality of life during treatment (24).

The study is limited by the fact that it only includes a small number of patients and a short follow-up after treatment. Thus, it can only serve as a preliminary report which warrant further validation in future prospective studies.

## Conclusion

Image-guided radiotherapy delivered with HT may potentially reduce the risk of radiation related severe toxicities, and especially lung toxicities in the setting of concurrent chemoradiation for limited-stage small cell lung cancer. This may allow for further radiation dose escalation to the gross tumor to improve treatment outcome. Prospective studies with a large number of patients should be conducted in the future to further validate this hypothesis.

## Conflict of Interest Statement

The authors declare that the research was conducted in the absence of any commercial or financial relationships that could be construed as a potential conflict of interest.

## References

[1] Scullier JP Lafitte JJ Efremidis A Florin MC Lecomte J Berchier MC A phase III randomized study of concomitant induction radiochemotherapy testing two modalities of radiosensitization by cisplatin (standard versus daily) for limited small-cell lung cancer. Ann Oncol (2008) 19:1691–7 10.1093/annonc/mdn354 18504252

[2] Bonner JA Sloan JA Shanahan TG Brooks BJ Marks RS Krook JE Phase III comparison of twice-daily split course irradiation versus once-daily irradiation for patients with limited stage small-cell lung carcinoma. J Clin Oncol (1999) 17:2681–91 10561342 10.1200/JCO.1999.17.9.2681

[3] Hu X Bao Y Zhang L Guo Y Chen YY Li KX Chemotherapy tumor extent for limited-stage small cell lung cancer. Cancer (2012) 118:278–87 10.1002/cncr.26119 21598237

[4] Colaco R Sheikh H Lorigan P Blackhall F Hulse P Califano R Omitting elective nodal irradiation in limited-stage small cell lung cancer-Evidence from a phase II trial. Lung Cancer (2012) 76:72–7 10.1016/j.lungcan.2011.09.015 22014897

[5] Pijls-Johannesma M De Ruysscher D Vansteenkiste J Kester A Rutten I Lambin P Timing of chest radiotherapy in patients with limited stage small cell lung cancer: a systemic review and meta-analysis of randomized controlled trials. Cancer Treat Rev (2007) 33:461–73 10.1016/j.ctrv.2007.03.002 17513057

[6] Takada M Fukuoka M Kawahara M Sugiura T Yokohama A Yokota S Phase III study of concurrent versus sequential thoracic radiotherapy in combination with cisplatin and etoposide for limited stage small cell lung cancer: results of the Japan Clinical Oncology Group Study 9104. J Clin Oncol (2002) 20:3054–60 10.1200/JCO.2002.12.071 12118018

[7] Spiro SG James LE Rudd RM Trask CW Tobias JS Snee M Early compared with late radiotherapy in combined modality treatment for limited disease small cell lung cancer: a London Lung Cancer Group Multicenter randomized clinical trial and meta-analysis. J Clin Oncol (2006) 24:3823–30 10.1200/JCO.2005.05.3181 16921033

[8] Nguyen NP Vock J Vinh-Hung V Ceizyk M Smith-Raymond L Stevie M Feasibility of image-guided radiotherapy based on helical tomotherapy to reduce contralateral parotid dose in head and neck cancer. BMC Cancer (2012) 12:175 10.1186/1471-2407-12-175 22578076 PMC3411401

[9] Nguyen NP Chi A Betz M Almeida F Vos P Davis R Feasibility of intensity-modulated and image-guided radiotherapy for functional organ preservation in locally advanced laryngeal cancer. PLoS One (2012) 7:e42729 10.1371/journal.pone.0042729 22916151 PMC3423414

[10] Nguyen NP Smith-Raymond L Vinh-Hung V Sloan D Davis R Vos P Feasibility of tomotherapy to spare the cochlea from excessive radiation in head and neck cancer. Oral Oncol (2011) 47:414–9 10.1016/j.oraloncology.2011.03.011 21474364

[11] Nguyen NP Ceizyk M Almeida F Chi A Betz M Modarresifar H Effectiveness of image-guided radiotherapy for locally advanced rectal cancer. Ann Surg Oncol (2011) 18:380–5 10.1245/s10434-010-1329-0 20848224

[12] Nguyen NP Vock J Sroka T Khan R Jang S Chi A Feasibility of image-guided radiotherapy based on tomotherapy for the treatment of locally advanced anal carcinoma. Anticancer Res (2011) 31:4393–6 22199304

[13] Turrisi AT Kim K Blum R Sause WT Livingston RB Komaki R Twice-daily compared with once-daily thoracic radiotherapy in limited small-cell lung cancer treated concurrently with cisplatin and etoposide. N Engl J Med (1999) 340:265–71 10.1056/NEJM199901283400403 9920950

[14] Hu X Bao Y Zhang L Guo Y Chen YY Li KX Omitting elective nodal irradiation and irradiating postinduction versus preinduction chemotherapy tumor extend for limited stage small cell lung cancer. Cancer (2012) 118:278–87 10.1002/cncr.26119 21598237

[15] Schild SE Bonner JA Shanahan TG Brooks BJ Marks RS Geyer SM Long-term results of a phase III trial comparing once-daily radiotherapy with twice daily radiotherapy in limited-stage small cell lung cancer. Int J Radiat Oncol Biol Phys (2004) 59:943–51 10.1016/j.ijrobp.2004.01.055 15234027

[16] Murray N Coy P Pater JL Hodson I Arnold A Zee BC Importance of timing for thoracic irradiation in the combined modality treatment of limited-stage small cell lung cancer. J Clin Oncol (1993) 11:336–44 8381164 10.1200/JCO.1993.11.2.336

[17] Gregor A Drings P Burghouts J Postmus PE Morgan D Sahmoud T Randomized trial of alternating versus sequential radiotherapy/chemotherapy in limited disease patients with small cell lung cancer. J Clin Oncol (1997) 15:2840–9 9256127 10.1200/JCO.1997.15.8.2840

[18] Bogard JA Herndon JE Lyss AP Watson D Miller AA Lee ME 70 Gy thoracic radiotherapy is feasible concurrent with chemotherapy for limited-stage small cell lung cancer: analysis of cancer and leukemia group B study 39808. Int J Radiat Oncol Biol Phys (2004) 59:460–8 10.1016/j.ijrobp.2003.10.021 15145163

[19] Bezjak A Rumble RB Rodrigues G Hope A Warde P Members of the IMRT indication panel. Clin Oncol (2012) 24:508–20 10.1016/j.clon.2012.05.007 22726417

[20] Shirvani SM Komaki R Heymach JV Fossela SV Chang JY Positron emission tomography/computed-tomography-guided intensity-modulated radiotherapy for limited-stage small cell lung cancer. Int J Radiat Oncol Biol Phys (2012) 82:91–7 10.1016/j.ijrobp.2010.12.072 21489716 PMC3465842

[21] Guckenberger M Wilbert J Richter A Baier K Flentje M Potential of adaptive radiotherapy to escalate radiation dose in combined radiochemotherapy for locally advanced non-small cell lung cancer. Int J Radiat Oncol Biol Phys (2011) 79:901–8 10.1016/j.ijrobp.2010.04.050 20708850

[22] Hugo GD Weiss E Badawi A Orton M Localization accuracy of the clinical target volume during image-guided radiotherapy of lung cancer. Int J Radiat Oncol Biol Phys (2011) 81:560–7 10.1016/j.ijrobp.2010.11.032 21277096 PMC3106135

[23] Song CH Pyo H Moon SH Kim TH Cho KH Treatment-related pneumonitis and acute esophagitis in non-small cell lung cancer patients treated with chemotherapy and radiotherapy. Int J Radiat Oncol Biol Phys (2010) 78:651–8 10.1016/j.ijrobp.2009.08.068 20207499

[24] Garces YI Okuno SH Schild SE Mandrekar SJ Bot BM Martens JM Phase I North Central Cancer Treatment Group trial N9923 of escalating doses of twice daily thoracic radiotherapy with amifostine and with alternating chemotherapy in limited stage small-cell lung cancer. Int J Radiat Oncol Biol Phys (2007) 67:995–1001 10.1016/j.ijrobp.2006.10.034 17336213

